# Self-reported social class in adolescents: validity and relationship with gradients in self-reported health

**DOI:** 10.1186/1472-6963-7-151

**Published:** 2007-09-24

**Authors:** María-Jesús Pueyo, Vicky Serra-Sutton, Jordi Alonso, Barbara Starfield, Luis Rajmil

**Affiliations:** 1 Institut Català de la Salut, Barcelona, Spain; 2 Agència per a la Qualitat, Recerca i Avaluació en Salut (AQuRA Salut, ex Agència d'Avaluació de Tecnologia i Recerca Mèdiques), Barcelona, Spain; 3 Institut Municipal d'Investigació Mèdica, Barcelona, Spain; 4 Department of Health Policy and Management, Johns Hopkins University, Baltimore, USA

## Abstract

**Background:**

Analyzing social differences in the health of adolescents is a challenge. The accuracy of adolescent's report on familial socio-economic position is unknown. The aims of the study were to examine the validity of measuring occupational social class and family level of education reported by adolescents aged 12 to 18, and the relationship between social position and self-reported health.

**Methods:**

A sample of 1453 Spanish adolescents 12 to 18 years old from urban and rural areas completed a self-administered questionnaire including the Child Health and Illness Profile-Adolescent Edition (CHIP-AE), and data on parental occupational social class (OSC) and level of education (LE). The responsible person for a sub-sample of teenagers (n = 91) were interviewed by phone. Kappa coefficients were estimated to analyze agreement between adolescents and proxy-respondents, and logistic regression models were adjusted to analyze factors associated with missing answers and disagreements. Effect size (ES) was calculated to analyze the relationship between OSC, LE and the CHIP-AE domain scores.

**Results:**

Missing answers were higher for father's (24.2%) and mother's (45.7%) occupational status than for parental education (8.4%, and 8.1% respectively), and belonging to a non-standard family was associated with more incomplete reporting of social position (OR = 4,98; 95%CI = 1,3–18,8) as was agreement between a parent and the adolescent. There were significant social class gradients, most notably for aspects of health related to resilience to threats to illness.

**Conclusion:**

Adolescents can acceptably self-report on family occupation and level of education. Social class gradients are present in important aspects of health in adolescents.

## Background

Measurement of social difference in health of adolescents presents challenges not present in the study of social gradients in health among adults. Reports of socio-economic position by adults are assumed to be accurate but the accuracy of child reports is unknown [1,2]. Indicators based on occupation, education or income have been used to assess socio-economic position [3,4]. With the mass entry of women into the labor market and with the emergence of new family structures, determining the socio-economic status of children and adolescents on the basis of the father's occupation may no longer be an adequate representation of family socioeconomic position. In self-administered adolescent questionnaires, up to 40% or responses on paternal occupation are invalid or missing, which has prompted the development of other measures of material well-being that are easier to elicit [5-7].

The accuracy of responses has been positively associated with age and the nearness in time and space with the parental characteristics being investigated [8]. The level of agreement between adolescents and proxy-respondents reporting education and occupation has been shown as moderate [9,10] improving with age, and worsening when the adolescent does not live with the parent about whom information is being asked. No gender differences were found.

In terms of analyzing social differences in health, the measurement of adolescent health is a challenge. In the last decade a number of questionnaires have been developed which attempt to measure self-perceived health and health-related quality of life, which have proved to be sensitive to social inequalities. The Child Health and Illness Profile – Adolescent Edition (CHIP-AE) [11] is a generic self-administered questionnaire developed in the United States and which has been adapted in Spain, with acceptable reliability and validity [12,13].

The objectives of this study were to examine the validity of measuring occupational social class (OSC) and family level of education (LE), reported by adolescents aged 12 to 18, and the relationship between social position and self-reported health.

## Methods

### Study design and sample selection

This study was carried out during the academic year 1999–2000 with ethical approval of the Research Committee of the School of Public Health of Catalonia. Before administering the questionnaires, consent to participate was asked to all selected schools. Letters were sent to parents with information about the study and to request their consent for their children to participate in the study. A sample of adolescents aged 12–18 was selected in secondary schools in an urban area (Barcelona) and a rural area (Piera) during the academic year 1999–2000 (n = 1774). The urban sample was selected using a two-stage sampling design. The schools were categorized in public or private/subsidized, and according to the Family Economic Capacity Index (ICEF) [14]. This aggregate index categorizes the socio-economic level of schools according to their neighborhood and was stratified in three levels (high, medium and low). In the following stage, school classes were chosen to include all educational levels in each stratum, and all students in each of the selected classes. The rural sample was chosen by systematic sampling of all adolescents of 12–18 enrolled at the two secondary schools in the village (one public and one private).

The instrument (CHIP-AE) was self-administered, and the research team and school staff organized the session to minimize disturbance of the school's educational program. Data was collected by a research team member (VSS) while the teacher was in the classroom most of the times.

The sub-sample for the study of agreement between adolescents and parents was selected from one public secondary school of a medium ICEF area in the urban area. Parents of the interviewed students (n = 160) were selected to take part in a telephone interview about socio-demographic data, including some questions from CHIP-AE. The interview was conducted during the same academic term as the adolescents' self-questionnaire. A minimum of 5 calls were made, at different times of day, to try to contact the parent primarily responsible for each adolescent.

### Measures

The socio-demographic variables analyzed were: age (12–15 and 16–18); sex; area (rural or urban), and the type of school (public and private/subsidized). Family type was analyzed on the basis of the number of people living in the house and relationship with the adolescent, in three groups: "standard"(the adolescent lived with both parents only); "single-parent" (living with one parent only), and "other" (other adults present). This characteristic was subsequently recoded into two: "standard family" as before, and "non-standard family" which includes the categories of "single-parent" and "other".

### Socio-economic variables

Information on the occupation of both parents was collected using an open question. One of the researchers (VSS) classified the responses into 9 categories according to the National Spanish Classification of Occupations, and after that occupational social class (OSC) was assessed by categorizing occupations according to the 5 categories (I–V) set out by the Spanish Society of Epidemiology [4] and later combined into three groups: classes I–II representing management staff in commerce and public administration and professions with university degrees; class III representing technical and support staff; and classes IV–V representing qualified and unqualified manual workers. The variables analyzed on OSC are: paternal, maternal and highest OSC, which reflects the highest social class within the family. The working situation of the father and mother separately were classified under 9 categories: full-time work, part-time work, unemployed, retired, receiving sickness benefit, student, deceased, not known and, in the case of women, the additional category of housewife. Family level of education (LE) was determined on the basis of the highest level completed by either the father or mother, in 6 categories subsequently reduced to 3: primary school or lower; secondary level (baccalaureate or technical training) and university qualifications. In accordance with recommendations to broaden the concept of socioeconomic position measurement, we also ascertained receipt of unemployment benefit, free school meals and other benefits was gathered through a yes/no question.

The variables collected in phone interviews with the parents included: highest maternal/paternal OSC; working situation of the father and mother; highest maternal/paternal LE; whether in receipt of free meals and other benefits. These variables were categorized in the same manner as for the sample of the adolescents.

### Health status measurement

The CHIP-AE questionnaire contains 183 questions divided in 6 domains and 20 sub-domains. Satisfaction covers satisfaction with health and self-esteem (12 items); Discomfort covers physical and emotional discomfort and limitations of activity (45 items); Resilience covers family involvement, social problem-solving, physical activity and home safety and health (31 items); Risks covers individual risks, threats to achievement and peer influence (38 items); Achievement covers academic and work performance (11 items); and Disorders contains a list of illnesses, injuries and impairments (45 items). The score of each sub-domain is obtained from the mean of the responses using a Likert type scale between 1 and 5; and each domain score is obtained from the mean score of its subdomains. In order to facilitate interpretation of the scoring, the domains have been standardized to an arbitrary mean of 50 and a standard deviation (SD) of 10, based on the individual score and the mean of the reference group. Mean scores for the Barcelona adolescents have been taken as the standard population [15]. Higher scores indicate better health in all domains.

### Statistical analysis

#### 1. Completeness and validity of responses

The responses "no answer" and "don't know" to the question about parent's working situation were analyzed together as the percentage of "don't know" was very low and there were no differences in terms of age, gender, type of family, ICEF, type of school area, nor in family level of education or reception of benefits, between adolescents who answered "don't know" and adolescents with missing values. The missing responses (no answer or don't know) in all variables of socio-economic level were analyzed in terms of age, sex, family type, LE, OSC, location, type of school and ICEF in the urban area. The factors associated with no-responses on OSC and LE were analyzed using multivariate logistic regression, controlling for the effect of socio-demographic variables. Response/no-response was considered as a dependent variable of different socio-economic variables (0 response, 1 missing or "don't know") and as a predictive variable for LE (in social class), OSC (in LE) and the domains Satisfaction, Discomfort, Resilience, Risks and Academic achievement of CHIP-AE categorized in 2 (0: score > percentile 25; 1: score ≤ p25).

The association between self-reported OSC and LE, and the type of school (public and private/subsidized), or ICEF (only in the urban sample) were analyzed by means of Chi Square.

The percentages of agreement and kappa coefficients [16] were estimated between the responses of the adolescents and those of the proxy-respondents to questions about OSC, education, and receipt of benefits (unemployment benefit, support or scholarships). The analysis of agreement was also stratified by age, sex and family type. Logistic regression models were adjusted taking as a dependent variable the agreement/disagreement of the responses of the adolescents and the informants about paternal, maternal and highest OSC, and paternal, maternal and highest LE, and as predictive variables age, sex and family type, as well as the domains of the CHIP-AE

#### 2. Health Status and socioeconomic position

The mean scores for the CHIP-AE domains in each category of OSC and LE were calculated, from which was obtained the effect size (ES) between groups as a standard measure of the relative size of the difference between the groups compared in pairs [17]. Following convention, values above 0.8 represent a high size effect, 0.5 – 0.8 a moderate effect, and between 0.2 and 0.49 low.

## Results

A total of 1453 adolescents was included in the final analysis (response rate = 82%, average age 14.9). 259 adolescents were absent on the day of questionnaire administration and in 62 cases, parents refused permission for their children to participate in the study. 60% were aged less than 16; 51.2% were boys; 60.1% came from the urban sample of which 17.3% lived in a neighborhood with a low ICEF (table 1). Eighty percent lived in a standard family with both parents. In 50.7% the highest level of education in the family was primary, and 54.5% belonged to the least privileged social class (IV–V).

**Table 1:** Socio-demographic and health indicators, and missing responses among adolescent sample and proxy sample

	Adolescent sample (n = 1453)			Proxy sample (n = 91)	
		**N %**	**Missing Responses N (%)**	**N %**	**Missing Responses N (%)**

Age	12 – 15	881 (60.6)	_	46 (50.5)	_
	16 – 18	572 (39.4)		45 (49.5)	
Sex	Male	752 (51.8)	_	48 (52.7)	_
	Female	701 (48.2)		43 (47.3)	
Number of persons in the household	<= 5	1295 (89.1)	11 (0.8)	81 (89)	2 (2.2)
	> 5	147 (10.1)		8 (8.8)	
Family type	Standard	1161 (79.9)	29 (2)	73 (80.2)	_
	Single-parent	120 (8.3)		15 (16.5)	
	Other	143 (9.8)		3 (3.3)	
Location	Urban	902 (62.1)	_	NA	
	Rural	501 (37.9)			
ICEF (only in the urban sample)	Low	251 (17.3)	_	NA	
	Medium	330 (22.7)			
	High	321 (22.1)			
Type of School	Public	836 (57.5)	_	NA	
	Private	617 (42.5)			
Unemployment benefit	Yes	96 (6.6)	310 (21.3)	7 (7.7)	_
	No	1047 (72.1)		84 (92.3)	
Food benefit	Yes	137 (9)	103 (7.1)	1 (1.1)	_
	No	1213 (83.5)		90 (98.9)	
Family benefit	Yes	72 (5)	278 (19.1)	2 (2.2)	_
	No	1103 (75.9)		89 (97.8)	
Level of paternal education	Primary	813 (55.9)	122 (8.4)	49 (53.8)	2 (2.2)
	Secondary	282 (19.4)		30 (33)	
	University	236 (16.2)		10 (11)	
Level of maternal education	Primary	952 (65.5)	118 (8.1)	48 (52.7)	3 (3.3)
	Secondary	251 (17.3)		30 (33)	
	University	132 (9.1)		10 (11)	
Highest family education level	Primary	737 (50.7)	83 (5.7)	39 (42.9)	_
	Secondary	351 (24.2)		34 (37.4)	
	University	282 (19.4)		18 (19.8)	
Paternal social Class	I – II	255 (17.5)	352 (24.2)	14 (15.4)	8 (8.8)
	III	263 (18.1)		20 (22)	
	IV – V	583 (40.1)		49 (53.8)	
Maternal social class	I – II	132 (9.1)	664 (45.7)	11 (12.1)	39 (42.9)
	III	193 (13.3)		14 (15.4)	
	IV – V	464 (31.9)		27 (29.7)	
Highest social class	I–II	181 (12.5)	204 (14)	12 (13.2)	3 (3.3)
	III	276 (19)		20 (22)	
	IV–V	792 (54.5)		56 (61.5)	
Paternal work	No	96 (6.6)	60 (4.1)	9 (9.9)	2 (2.2)
	Yes	1297 (89.3)		80 (87.9)	
Maternal work	No	472 (32.5)	35 (2.4)	39 (42.9)	1 (1.1)
	Yes	946 (65.1)		51 (56)	

NA: Not applicable

Responses were received from 91 of the 160 proxy-respondents (response rate = 56%). Mothers were the principal informants (83/91). Distribution of socio-demographic variables for the sub-sample of adolescents whose proxy-respondents were interviewed and the rest of the adolescents were similar, except that there were a higher proportion of families with a secondary level of education and with paternal OSC IV–V in the sub-sample of adolescents whose parents participated in the study. No differences were found in adolescents' health status between participants and non-participants.

### 1. Completeness and validity of responses

Paternal and maternal OSC was missing in 24.2% and 45.7% of adolescent reports (table 1). Table 2 shows the logistic regression models of the non-responses on socio-economic variables adjusted by age, sex, location and family type. The probability of a non-response on paternal LE was higher when the adolescent was younger (OR = 4.28, 95%CI = 2.46 – 7.45). Factors associated with non-response on maternal LE were age less than 16 (OR = 6.95, 95%CI = 3.61 – 13.36), and male gender (OR = 1.63, 95%CI = 1.07 – 2.47). Non-response on paternal OSC was associated with belonging to a non-standard family (OR=2.48; 95%IC 1.85-3.32). In contrast, non-response on maternal OSC was associated with being over 16 (OR = 0.74; 95%CI = 0.59 – 0.94), masculine gender (OR = 1.41; 95%CI = 1.12 – 1.74), and living in the rural area (OR = 1.46; 95%CI = 1.16 – 1.84). Non-response on the highest OSC was associated with being a boy (OR = 1.41; 95%CI = 1.02 – 1.95), belonging to a non-standard family (OR = 1.89; 95%CI = 1.32 – 2.72), and living in the rural area (OR = 1.57; 95%CI = 1.13 – 2.19).

**Table 2:** Logistic regression equations of non-responses on variables of socio-economic position. OR (95%CI)

		No response on paternal education level	No response on maternal education level	No response on highest education level	No response on paternal social class	No response on maternal social class	No response on highest social class
Age	>15	1^a^	1^a^	1^a^	1^a^	1^a^	1^a^
	12–15	**4.28 (2.46 – 7.45)**	**6.95 (3.61 – 13.4)**	**6.24 (2.9 – 13.4)**	1.04 (0.79 – 1.38)	**0.74 (0.59–0.94)**	0.99 (0.70 – 1.41)
Sex	Female	1^a^	1^a^	1^a^	1^a^	1^a^	1^a^
	Male	**1.57 (1.04 – 2.38)**	**1.63 (1.07 – 2.5)**	**1.75 (1.07 – 2.86)**	1.13 (0.87 – 1.47)	**1.4 (1.12 – 1.74)**	**1.41 (1.02 – 1.95)**
Family type	Standard	1^a^	1^a^	1^a^	1^a^	1^a^	1^a^
	Non-standard^b^	**3.1 (2.02 – 4.75)**	1.32 (0.81 – 2.13)	**1.9 (1.12 – 3.2)**	**2.48 (1.85 – 3.32)**	0.88 (0.67 – 1.16)	**1.89 (1.32 – 2.72)**
Location	Urban	1^a^	1^a^	1^a^	1^a^	1^a^	1^a^
	Rural	0.74 (0.49 – 1.1)	0.66 (0.43 – 1.02)	0.71 (0.43 – 1.17)	1.21 (0.93 – 1.59)	**1.46 (1.16 – 1.84)**	**1.57 (1.13 – 2.19)**
Resilience	High (>p25)	1^a^	1^a^	1^a^	1^a^	1^a^	1^a^
	Low (≤p25)	1.19 (0.73 – 1.94)	1.57 (0.95 – 2.6)	1.55 (0.85 – 2.79)	1.26 (0.92 – 1.73)	0.92 (0.71 – 1.19)	1.29 (0.87 – 1.93)
Academic achievement	High (>p25)	1^a^	1^a^	1^a^	1^a^	1^a^	1^a^
	Low (≤p25)	1.00 (0.63 – 1.59)	1.22 (0.77 – 1.93)	1.16 (0.67 – 1.98)	1.1 (0.83 – 1.46)	1.01 (0.79 – 1.29)	0.92 (0.64 – 1.32)
Risks	High (>p25)	1^a^	1^a^	1^a^	1^a^	1^a^	1^a^
	
	Low (≤p25)	0.75 (0.45 – 1.26)	0.56 (0.3 – 1.007)	0.54 (0.27 – 1.06)	1.17 (0.87 – 1.57)	0.92 (0.71 – 1.19)	1.19 (0.82 – 1.72)

The dependent variables have been calculated as response = 0, no response = 1; ^a^Reference category; ^b^The category of "non-standard families" comprises both "single parent" and "other" families. The CHIP-AE domains have been introduced as categorical variables. Higher score signifies better health. Each model is adjusted for the rest of the variables in the table.

**Figure 1:**
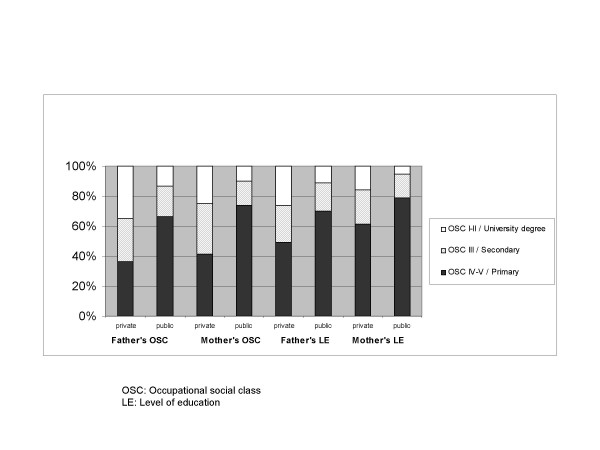
Distribution of parents' occupational social class and level of education by the type of school.

Figure 1 shows the distribution of parental occupational social class and level of education by the type of school. Adolescents in private schools reported higher percentage of paternal and maternal occupational social class I–II than adolescents in public schools (35% vs 12%, p < 0.05 for paternal OSC). Adolescents in private schools also reported higher percentage of paternal and maternal university degree than adolescents in public schools (15% vs 5%, p < 0.05 for maternal LE). Similar results were found in the urban sample comparing OSC and LE by ICEF (data not shown).

The percentages of agreement between adolescents and their parents in the urban sample ranged from 87.7% for paternal OSC to 63.6% for highest LE (table 3). The kappa values were lower for LE (kappa range = 0.39 – 0.51) than for OSC (kappa range = 0.52 – 0.77). No differences in agreement was found after stratifying by age, sex and family type. Disagreement on paternal OSC was greater in non-standard families (OR = 4.9; 95%CI = 1.3 – 18.8), a finding similar to that of the highest OSC (OR = 4.63; 95%CI = 1.11 – 19.3) (table 4).

**Table 3:** Percentage of agreement and kappa coefficient (95%CI) between the adolescents' responses and those of their proxy-respondents

	% of agreement	Kappa coefficient	(95%CI)
Paternal level of education	67.1	0.39	(0.23 – 0.54)
Maternal level of education	73.8	0.51	(0.36 – 0.67)
Highest level of education	63.6	0.41	(0.27 – 0.56)
Paternal social class	87.7	0.77	(0.58 – 0.96)
Maternal social class	74.4	0.56	(0.33 – 0.78)
Highest familiar social class	76.3	0.52	(0.35 – 0.68)

Paternal, maternal and highest level of education are stratified in 3 categories: primary, secondary, and university degree. Paternal, maternal, and highest social class in 3 categories: I–II, III, IV–V. Subsample of adolescents and proxy-respondents within the urban sample.

**Table 4:** Logistic regression models of the non-agreement between adolescents and proxy respondents (95%CI) in responses on different socio-economic variables

		Non-agreement on paternal education	Non-agreement on maternal education	Non-agreement on highest education	Non-agreement on paternal OSC	Non-agreement on maternal OSC	Non-agreement on highest OSC
Age	> 15	1^a^	1^a^	1^a^	1^a^	1^a^	1^a^
	12–15	0.83 (0.3 – 2.2)	0.47 (0.2 – 1.4)	0.76 (0.3 – 2.0)	0.73 (0.3 – 2.0)	0.44 (0.2 – 1.2)	0.38 (0.1 – 1.2)
Sex	Male	1^a^	1^a^	1^a^	1^a^	1^a^	1^a^
	Female	1.30 (0.5 – 3.3)	1.2 (0.4 – 2.9)	0.90 (0.3 – 2.3)	2.6 (0.9 – 6.9)	0.95 (0.4 – 2.5)	1.22 (0.4 – 3.5)
Family type^b^	Standard	1^a^	1^a^	1^a^	1^a^	1^a^	1^a^
	Non-standard	1.15 (0.3 – 3.9)	2.28 (0.6 – 7.8)	0.63 (0.2 – 2.2)	**4.98 (1.3 – 18.8)**	3.0 (0.9 – 10.4)	**4.63 (1.1 – 19.2)**
Resilience	High (>p25)	1^a^	1^a^	1^a^	1^a^	1^a^	1^a^
	Low (≤p25)	0.65 (0.2 – 1.8)	1.25 (0.4 – 3.7)	0.95 (0.3 – 2.7)	1.31 (0.4 – 4.01)	0.69 (0.2 – 2.09)	0.85 (0.2 – 3.01)
Risks	Low (>p25)	1^a^	1^a^	1^a^	1^a^	1^a^	1^a^
	High(≤p25)	0.62 (0.2 – 1.8)	0.6 (0.2 – 1.9)	0.88 (0.3 – 2.6)	0.67 (0.2 – 2.1)	0.97 (0.3 – 2.8)	0.64 (0.2 – 2.2)
Academic achievement	High (>p25)	1^a^	1^a^	1^a^	1^a^	1^a^	1^a^
	Low (≤p25)	2.01 (0.6 – 6.2)	1.69 (0.5 – 5.6)	2.22 (0.7 – 6.8)	0.72 (0.2 – 2.2)	1.04 (0.3 – 3.2)	1.1 (0.3 – 3.8)

The dependent variables have been calculated as: 0 = agreement between adolescents and proxies, 1 = non-agreement. ^a ^Reference category. ^b ^The category of "non-standard families" comprises both "single-parent" and "other" families. Each model is adjusted for the rest of variables in the table. Higher scores in the CHIP-AE domains means better health (better Resilience and academic achievement, and low Risks)

### 2. Relationship between socio-economic status and health

Higher scores in the domains of Satisfaction and Discomfort were found among boys and in adolescents with 2-parent standard families with a low effect size (table 5). No differences were found between the score in these domains and the LE and OSC. Differences were observed in the Resilience domain according to the LE (paternal, maternal and highest), with low effect size between the extremes (primary level vs. university degree). A similar finding was observed with the OSC, with the highest scores in the more advantaged social classes (I–II), and low effect size between the extremes (I–II vs. IV – V), for paternal OSC (effect size = 0.3), maternal OSC (effect size = 0.44). In the case of maternal OSC, a difference between social classes I – II and III was also observed. In the Risk domain, significant differences were noted according to the age of the adolescent, with lower scores in older respondents, and a high ES (0.87). Adolescents from non-standard families presented worse scores in the Risk domain, with a low effect size. Children of parents with secondary LE presented a lower (worse) score in Risks (ES = 0.23) compared to children of parents with university qualifications. For adolescents over 15, the score in the sub-domain of academic achievement was lower than that of those under 15, with an effect size = 0.47. Differences were also noted in academic achievement, with highest scores in those from families with a high LE (whether paternal, maternal or highest) and where the maternal OSC was I – II, in both cases with a low effect size.

**Table 5:** Effect size between categories of socio-economic variables and CHIP-AE domains

Variable		Satisfaction		Discomfort		Resilience		Risks		Academic achievement	
		
		M	Effect size	M	Effect size	M	Effect size	M	Effect size	M	Effect size
Sex	Female	47.1	0.48	48	0.48	47.9	0.28	51.3		51.1	
	Male	52.7		52.5		50.7		50.2		49.6	
Age	12–15	51.0	0.24	51.3	0.24	49.4		53.9	0.87	52.2	0.47
	>15	48.4		48.9		49.3		46		47.6	
Type of family	Standard	50.5	0.24	50.8	0.21	50	0.33	51.3	0.26	50.7	
	Non-standard	48.1		48.7		46.7		48.7		48.9	
Paternal level of education	University	50.8		50.1		51.3		51.5	0.23	52.4	0.25
	Secondary	49.8		50.8		50.2		49.2		49.8	
	Primary	49.6		50.1		48.6	0.27	50.4		50	0.25
Maternal level of education	University	50.5		50.6		52.3		51.7		52.4	
	Secondary	50.5		49.6		50.6		49.7		51.3	
	Primary	49.7		50.4		48.7	0.36	50.2		49.8	0.27
Highest level of education	University	50.6		50.2		51.1		51.5		52.3	0.23
	Secondary	49.7		50.1		49.9		49.3		49.9	
	Primary	49.7		50.4		48.5	0.27	50.5		49.8	0.26
Paternal social class	I–II	50.7		50.8		51.8		50.3		50.7	
	III	50.1		49.9		50.7		50.2		50.1	
	IV–V	49.9		50.4		48.9	0.30	51.5		49.9	
Maternal social class	I–II	50.1		49.9		52.5	0.29	51.9	0.24	53.3	0.24
	III	49.3		49.4		49.8		49.5		50.7	
	IV–V	49.2		51.0		48.1	0.44	50.7		49.9	0.34
Highest social class	I–II	50.7		51.3		52.4		50.6		51.9	
	III	50.3		49.5		50.5		49.3		50.9	
	IV–V	49.8		50.4		48.8	0.36	51.3		49.9	

The table shows ES > 0,2. Where there are 3 categories, the first box indicates the EFFECT SIZE between the first and second categories; the second is that between the second and third categories, and the third box that between the first and the third categories. Higher scores in the CHIP-AE domains mean better health: high Satisfaction, better Resilience and academic achievement, and less Discomfort and Risks

## Discussion

The present study has attempted to help researchers to decide on which indicator of socioeconomic position could be used in adolescents, and to analyze the influence of socioeconomic position on self-perceived health. Occupational social class is an indicator widely used in adults while adolescents are in general excluded from this analysis.

Although one in seven adolescents didn't allow to assign the family's occupational social class, overall, adolescents' reports provide valid information about their family socioeconomic status. The response to the question about the LE of both parents was more exhaustive, but with a low level of agreement between adolescents and their mothers, when compared with the OSC.

The percentage of missing responses about parental OSC and LE are similar to those of other studies [9,10]. Girls answered more exhaustively than boys, although for those over 15 this only happened in relation to questions covering LE. As a consequence, younger adolescents could answer questions about OSC in an acceptable manner. Questions about OSC presented more missing responses, although the calculation of the variable on family OSC could still be determined in 86% of cases. Non-standard families were associated with a higher frequency of missing response about paternal OSC and LE, which indicates the importance of living with the parent about whom information is being collected.

In this study, contrary to the finding of Wardle [7], there was no relation between the economic level of the urban area where the school was located, as measured by the ICEF, and missing answers to the questions on OSC and LE. The kappa values found in this study were above 0.50 except for the question about paternal and highest education in the family. These values were similar to those found by Lien [9] and somewhat lower than those of Ensminger [10]. In the present study the agreement between adolescents and mothers was not influenced by the age or sex of the adolescent, although the type of family influenced the agreement on the paternal OSC. Overall, the percentage of agreement about occupation were higher than that about other family characteristics such as the presence of smokers in the family, the presence of physical or emotional problems, or the declaration about lifestyle and diet (data not shown). From this it may be concluded that adolescents are better informants about OSC than about other characteristics of family life.

Adolescents under-estimated the parental LE (for the adolescents 70.3% of mothers had only primary education, vs. 52.7% for proxy-respondents; and 69.2% of fathers against 53.8%, respectively), which in part explains the low level of agreement compared with that of occupation. This finding would tend to support the theory of Looker that adolescents respond better about parental characteristics that are near in time, such as occupation, than about more distant ones. Changes in administrative structure, and academic reforms that have occurred in Spain in the past could also influence the lower validity of the answers about LE.

The most valid indicator of socio-economic position collected in this study seems to be paternal OSC, which presents high kappa values and is little influenced by socio-demographic factors which affect the adolescents' answers. However, due to the completeness of responses and a moderate kappa value, maternal LE could be a good indicator of those aspects of socio-economic position that also influence the self-perceived health of adolescents. For the purpose of collecting information about socio-economic position in adolescents it could be recommended to use father's occupational social class as a first choice and maternal level of education as the second.

The moderate social gradient of some aspects of self-perceived health among adolescents was found. This finding is consistent with the studies of others[10,18] although the US studies found a clear gradient in the Satisfaction and Discomfort domains.

Family type was remarkably important in terms of the relation between socio-economic status and self-perceived health, in addition to the known differences by gender and age. Those from standard families presented better scores in all the CHIP-AE domains and sub-domains, similar results to the USA studies [10,18]. The absence of gradient in Satisfaction and Discomfort domains might be due to the difference in the percentage of adolescents in the Spanish sample living with both parents (80% in the present study vs. 47% in the Ensminger study). Furthermore, single-parent families showed a significantly higher percentage of mothers with university studies, living in urban areas with a high ICEF, which differs from the American sample. The Resilience domain was the most sensitive to differences in OSC and LE, with a significant gradient in the majority of the sub-domains. In the Risks domain, differences were observed between adolescents from families with university qualifications (maternal, paternal or highest) and the rest, although the worst scores were for families with secondary education. Adolescents from families with a better socio-economic position, especially those with a high LE, generally presented more protective factors and lower level of risks than for other families. A social gradient was also noted in academic performance. Better academic achievement in high socio-economic position adolescents indicates the high likelihood of inter-generational transmission of social position. Paternal, maternal and highest LE showed a statistically significant relationship in more domains than the OSC gradient. These results support the theory that social and human capital are both important factors in developing a healthy life[10].

The main limitation of this study is the small sample size in the sub-sample of adolescents and proxies, although it has been possible to establish comparisons between the indicators reported on by adolescents and their mothers. No statistically significant differences were found comparing data of adolescents whose parents did not participate with that of those who took part in the study, except for a higher participation by families with maternal secondary LE. We were not able to analyze criterion validity, as it would have been necessary to collect information on self-reported OSC and LE of both parents to obtain a gold-standard with which to compare the answers from the adolescents. Furthermore, in the current study the kappa values may have been influenced by the high prevalence of the level of primary education in the sub-sample [19]. Most cross-sectional studies are conducted in school settings, and adolescents are the only source of information, while parents are rarely involved the opportunity to study. Therefore, socioeconomic gradients in health and health services would be lost in these studies unless we rely on self-reported data by adolescents. Nevertheless, it is worth noting that information from both adolescents and their parents should be collected whenever this would be possible. The present analysis does not allow us to know to what extent missing answers could be from adolescents who avoid answering what they might consider embarrassing replies [8]. Nevertheless, this factor may have little influence on the results, given that other sections of the questionnaire with less percentage of missing answers can be considered more embarrassing than the socioeconomic status (e.g. risky and sexual behaviours).

The absence of significant differences in the domains of Satisfaction and Discomfort could be related to specific characteristics of the sample, particularly the small number of individuals in some cells means. That is, gradients were observed but the number of observations could have been too small to achieve significance. Finally, it should be noted that ethnic and family composition was more homogenous when the questionnaire was administered than currently in Spain. This is due to the fact that in recent years the percentage of immigration in Europe in general and Spain in particular has increased, as the percentage of different types of family. It may be that more recent administration of the survey would result in different findings.

## Conclusion

Adolescents seem to be acceptable informants on family OSC and LE. Social gradient in health also exists among adolescents. Health policy measures should take into account social class inequalities in health during adolescence.

## Competing interests

The author(s) declare that they have no competing interests.

## Authors' contributions

LR, VSS, BS and JA conceived the study, and participated in its design and coordination. MJP, VSS and LR participated in the protocol development and analysis of data. MJP wrote the first draft of the manuscript, and the rest of authors made substantial contributions to the interpretation of data, revised critically all the previous versions of the manuscript, and approved its final version.

## Pre-publication history

The pre-publication history for this paper can be accessed here:


